# Drosophila Models of Proteinopathies: the Little Fly that Could

**DOI:** 10.2174/138161212799315894

**Published:** 2012-03

**Authors:** Diego E Rincon-Limas, Kurt Jensen, Pedro Fernandez-Funez

**Affiliations:** 1 Departments of Neurology, McKnight Brain Institute, University of Florida, Gainesville, FL 32610, USA; 2 Departments of Neurosciences, McKnight Brain Institute, University of Florida, Gainesville, FL 32610, USA

**Keywords:** Drosophila models, neurodegeneration, protein misfolding, amyloids, Alzheimer, Parkinson, Huntington, Prion.

## Abstract

Alzheimer’s, Parkinson’s, and Huntington’s disease are complex neurodegenerative conditions with high prevalence characterized by protein misfolding and deposition in the brain. Considerable progress has been made in the last two decades in identifying the genes and proteins responsible for several human ‘proteinopathies’. A wide variety of wild type and mutant proteins associated with neurodegenerative conditions are structurally unstable, misfolded, and acquire conformations rich in ß-sheets (ß-state). These conformers form highly toxic self-assemblies that kill the neurons in stereotypical patterns. Unfortunately, the detailed understanding of the molecular and cellular perturbations caused by these proteins has not produced a single disease-modifying therapy. More than a decade ago, several groups demonstrated that human proteinopathies reproduce critical features of the disease in transgenic flies, including protein misfolding, aggregation, and neurotoxicity. These initial reports led to an explosion of research that has contributed to a better understanding of the molecular mechanisms regulating conformational dynamics and neurotoxic cascades. To remain relevant in this competitive environment, *Drosophila* models will need to expand their flexible, innovative, and multidisciplinary approaches to find new discoveries and translational applications.

## 1 INTRODUCTION

A number of human neurodegenerative conditions are associated with abnormal protein deposition in brain neurons. This group of *protein misfolding* disorders or *proteinopathies* includes some of the most common diseases among the elderly (e.g., Alzheimer’s [AD] and Parkinson’s [PD] disease), several dominantly inherited diseases (e.g., Huntington’s disease [HD], Spinocerebellar ataxias [SCA] 1 and 3), the aggressive amyotrophic lateral sclerosis (ALS) or Lou Gehrig’s disease, and the rare and unique Prion diseases (PrD). These protein misfolding disorders belong to a larger class of neurodegenerative diseases characterized by late onset, progressive loss of brain neurons that includes dominantly inherited RNA diseases (e.g., myotonic dystrophy, SCA8 and 10) and recessive loss-of-function disorders (e.g., Fragile X syndrome, Friedreich’s ataxia), among others. The proteinopathies constitute a heterogeneous group of brain disorders (and other systemic conditions that will not be reviewed here) that includes dominantly inherited disorders (AD, PD, HD, SCAs, ALS, PrD), sporadic (idiopathic) conditions (AD, PD, ALS, PrD) and infectious diseases (PrD). Some of the disorders have multiple etiologies, which contributed to the identification of the genes and molecular mechanisms that cause them. In the familial forms of the diseases, the mutant genes encode the proteins that aggregate in the sporadic forms of each disease, thus identifying the culprits in all forms of the disease. These proteins become structurally unstable upon mutation (Ataxin1 [Atx1], Cu/Zn superoxide dismutase 1), after abnormal proteolytic cleavage (Amyloid-ß42 [Aß42], Huntingtin [Htt]) or are naturally unstable in their normal state (Prion protein [PrP], α–Synuclein [α–Syn]). Regardless of the origin of the structural perturbations in these proteins, the conformational changes (misfolding) expose hydrophobic residues and increase the ß-sheet content (ß-state). The ß-state is unstable as a monomer because of the need to hide the exposed hydrophobic residues, leading to self-assembly into small, soluble aggregates (oligomers). These soluble assemblies are highly toxic in cell culture models and are proposed to be the neurotoxic species [1, 2]. Over time, oligomers aggregate by a “seeding-nucleation” model [3] into larger, insoluble, fibrillar structures that are detected by traditional histo-pathological techniques: amyloid plaques and neurofibrillary tangles in AD, Lewy bodies in PD, and nuclear inclusions in HD and SCAs. A testament to the complexity of these disorders is that two decades of tremendous advances in understanding the structural dynamics and biological properties of these proteins have provided no disease-modifying therapies. 

**Fig. (1):**
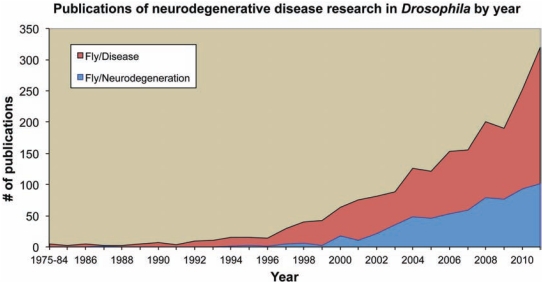
**The upward trend of disease model publications using flies.** Publications displayed in PubMed with general *disease* application (red) and those relevant to *neurodegenerative diseases* (blue). The numbers for 2011 are projections based on year-to-date publications. Search parameters: Blue: (neurodegenerative OR neurodegeneration) AND drosophila; Red: (disease OR neurodegenerative OR neurodegeneration) AND drosophila. Search terms were limited to the title and abstract to eliminate false positives. This can lead to underestimation of the number of papers (false negatives), but a brief visual analysis confirmed the sensitivity of the search.

Protein misfolding is mostly an intrinsic property of these pathogenic proteins: their amino acid sequences (either wild type or mutant) contain the information that makes them structurally unstable and capable of populating the ß-state [3]. Given the intrinsic structural instability and neurotoxicity of these proteins, it is not surprising how easy it was to replicate those properties in transgenic flies and how popular these disease models have become in the last decade. Two breakthrough reports in 1998 paved the way by modeling two dominant conditions in flies, HD and SCA3 [4, 5]. Several more papers came shortly after from laboratories that worked independently on the same idea [6-9], and an explosion of papers followed in the next decade (Fig. **1**). Here we review some of the most important contributions of *Drosophila* to unraveling the molecular mechanisms of neurodegeneration and discuss its future applications in gene and drug discovery efforts.

## 2 THE MIGHTY LITTLE FLY

*Drosophila melanogaster* is a small fruit fly with worldwide distribution that poses no threat to human health and agriculture. What made *Drosophila *attractive to T.H. Morgan in the early 1900s is that fruit flies have a short life cycle (10 days at 25°C) and produce a large progeny (upwards of 100 eggs per female). The pioneering work of Morgan and Sturtevant with these elegant, golden-colored flies produced the first mutation in 1906, *white*, which eliminated the pigment from the large eyes, and many more mutations followed in the ensuing decades. Many additional reasons make *Drosophila* the model of choice to tackle relevant biological problems: access to large collections of mutant strains, sophisticated genetic techniques, multiple approaches for manipulating gene expression, easy transgenesis, and compatibility with low- to mid-throughput screening [10]. Additionally, *Drosophila* has been a leader in genome sequencing and annotation [11]. 

*Drosophila *has been a favored tool for genetic studies for over 100 years and is an excellent model system to study a variety of biological processes, including development, signal transduction, cell biology, and immunity. Moreover, *Drosophila* has provided substantial contributions to fundamental questions in neurobiology: nervous system organization and function, information integration and processing, wiring and physiology of neural circuits, neurodegeneration, and the genetic control of behavior, sleep, memory, aggression, mating, and addiction [12]. A transformative moment in *Drosophila* research came with the realization of the extraordinary conservation of gene sequence/function with humans. In fact, some of the most important genes with key regulatory functions were first described in flies [see [12] for historical context], including Homeotic/HOX, decapentaplegic/TGF-ß, Wnt/wingless, Hedgehog/Sonic hedgehog. Indeed, over 75% of the human genes implicated in disease display highly significant sequence conservation at the protein level with *Drosophila* genes [13]. This high degree of functional conservation suggests that diseases resulting from disruption of conserved cellular pathways should be easily recapitulated at genetic and molecular levels in fruit flies.

## 3 THE EVER-EXPANDING TOOLBOX

*Drosophila *still maintains a healthy edge over other animal models in the ability to manipulate gene expression and function. The genetic toolbox in *Drosophila* is unrivaled due to constant innovation and the selfless sharing of ideas and resources [10, 14]. This is a brief summary of the most relied upon genetic tools in flies.

*Gene expression*. Following the recombinant DNA revolution of the 80s, which introduced the technology for incorporating transgenes in flies (always first!) and other animals [15], the early 90s brought another game changer: the UAS/Gal4 expression system. The heterologous UAS/Gal4 system imported from yeast is a binary expression system: one strain carries a transgene under the control of the UAS (upstream activating sequence) promoter sequence, and a second strain expresses the transcription factor Gal4 [16]. Transgenes are cloned into a freely available vector (pUAST) downstream of the UAS, followed by microinjection of embryos (now outsourced to commercial services). To induce transgene expression, the UAS-transgene strains are combined with strains expressing Gal4 under the control of highly diverse promoters (from ubiquitous to single cell), offering unparalleled experimental flexibility (Table **1**). The UAS/Gal4 system is a terrific tool for directing the spatial expression of transgenes; unfortunately, it provides poor temporal control. The introduction of the tripartite TARGET (temporal and regional gene expression targeting) system finally provided good temporal control to the UAS/Gal4 system, thus taking full advantage of the tens of thousand of existing UAS-regulated transgenes [17]. TARGET introduces a temperature-sensitive allele of the Gal4 inhibitor Gal80, which regulates gene expression by shifting from permissive temperature (19°C, Gal80^TS^ active, Gal4 inactive) to restrictive temperatures (30°C, Gal80^TS^ inactive, Gal4 active). TARGET has become a wonderful tool for manipulating critical processes in adult flies, particularly those associated with brain function, without affecting central nervous system (CNS) development.

*Mutant strains*. In addition to the classic mutations generated by chemicals and ionizing radiations, transposable elements (P-elements) have played a key role in the relentless push to mutate every gene in the fly genome (Table **1**). P-element-mediated gene discovery has introduced transposable elements near or inside 65% of *Drosophila* genes, making them available for further modification, including excision, *in vivo* tagging, and enhancer capture for Gal4 expression [10, 14]. Also, modified P-elements carrying the UAS enhancer with a minimal promoter allows misexpression of nearby genes, which have proved very useful in genetic screens [18]. Several versions of this transposon have been randomly inserted throughout the *Drosophila* genome, resulting in tens of thousands of invaluable strains stored in various stock centers (Table **1**). Moreover, the* Drosophila* field has fully incorporated the powerful RNA interference (RNAi) technology from *C. elegans* [19]. Researchers now have access to genome-wide collections of RNAi molecules (double stranded RNA) for cell culture assays plus three independent genome-wide collections of transgenic flies carrying RNAi constructs under the control of UAS (Table **1**). P-element technology has finally allowed the creation of a genome-wide collection of molecularly defined deletions, an invaluable resource for gene mapping. 

*Latest technologies*. The fly genome allows the most sophisticated manipulations of any animal model, from random transposon tagging to site-specific transgenesis. Latest efforts have focused on making gene targeting more efficient in *Drosophila* through the use of zinc-finger nucleases [20]. Also, recombination-mediated genetic engineering or recombineering, coupled with the bacteriophage φC31 integrase, has provided a new platform for easy and speedy manipulation of DNA fragments larger than 130,000 bases into the fly genome [14]. Finally, recent methods for the automated mass-injection of fly embryos will result in an expanded ability to test and use sophisticated P-element vectors and RNAi [21, 22]. This area of* Drosophila* research is very hot and continues to provide revolutionary technology every year.

## 4 FLY MODELS OF NEURODEGENERATIVE DISORDERS

The innovative models of human neurodegenerative diseases that appeared between 1998 and 2000 inspired a new field of research and created a whole generation of researchers dedicated to understand human neurological diseases using *Drosophila*. This community has produced hundreds of research papers in the last few years and is projected to publish more than 100 papers in 2011 (Fig. **1**). Current examples of *Drosophila* models of neurodegenerative disease include AD, PD, tauopathies, several polyglutamine disorders (HD, SCA1, SCA3 [a.k.a. Machado-Joseph disease], Spinobulbar muscular atrophy), ALS, PrD, dystonia, non-coding expansions (SCA8, myotonic dystrophy), and several recessive disorders, including Fragile-X syndrome and Friedreich’s ataxia, among others. These models have been reviewed recently in excellent papers and the reader should refer to them for more details [23, 24]. We next present a brief overview of the proteinopathies and how *Drosophila* has contributed to their understanding.

### 4.1 Alzheimer’s disease and Frontotemporal dementias

AD is the most prevalent neurodegenerative disorder affecting 1% of those 65 years old or older and up to 35% of those older than 85. With the rising life expectancy in advanced economies, AD is acquiring the dimensions of an epidemic [25]. AD first manifests by short-term memory loss, progressing to loss of executive functions and full dementia over several years. Upon autopsy, the AD brain is characterized by degeneration of the cortex and hippocampus, and by two types of protein deposits: extracellular amyloid plaques rich in the Aß42 peptide and intracellular neurofibrillary tangles (NFT) containing the microtubule associated protein Tau. According to the “amyloid hypothesis”, accumulation of the Aß42 peptide initiates the pathogenic cascade in AD, including Tau hyperphosphorylation, aberrant cellular signaling and, ultimately, cell death [26]. The Aß42 peptide is the result of ß- and γ-secretase cleavage of the transmembrane Amyloid Precursor Protein (APP). Mutations associated with familial AD affect APP and two proteins with γ-secretase activity, Presenilin 1 and 2, supporting the hypothesis that overproduction of Aß42 initiates AD pathogenesis. Only temporary symptomatic treatment for early AD is currently available, but the most promising upcoming therapies may be based on immunodepletion of specific conformers of Aß oligomers [27].

The first AD-related studies in flies focused on APP and showed that overexpression of *Drosophila* APPL (APP-Like) and human APP (Table **2**) cause axonal transport defects [28, 29]. This axonal dysfunction phenotype was also associated with other disease-related proteins (mutant Huntingtin), hinting at a common mechanism of neurodegeneration in different diseases [30]. Specific APP cleavage is key for accumulation of Aß42 and AD pathogenesis; interestingly, *Drosophila* possesses all the components necessary for both amyloid and non-amyloid processing of APP [31, 32]. The γ-secretase complex is essentially the same in flies and can cleave either APP or APPL at the γ-site in the transmembrane domain [31]. In addition, a *Drosophila* ß-secretase-like enzyme (dBACE) cleaves APPL (but not APP) in the ß-site, leading to the production of Aß42, formation of amyloid fibers and neurotoxicity [32]. The conservation of the amyloidogenic and non-amyloidogenic pathways in *Drosophila* created ideal conditions for further uncovering new genetic and pharmacologic regulators of APP processing. Although flies do not produce Aß42 naturally, overexpression of human APP and BACE combined with* Drosophila* Presenilin (Psn) results in Aß42 production and neurotoxicity [33]. Using these flies, a ß-secretase inhibitory peptide demonstrated increased potency when anchored to the membrane by a sterol moiety [34]. Moreover, known inhibitors of human Presenilins rescue the toxicity of triple transgenic flies and induce Notch-related developmental defects, indicating that these drugs interact with and inhibit *Drosophila* Psn [33, 35]. These observations suggest that fruit flies can be used for *in vivo* validation and toxicity evaluation of novel γ-secretase inhibitors of high importance for the pharmaceutical industry. In addition to these pharmacological applications, *Drosophila* models of AD have been applied to the discovery of novel genetic regulators of APP processing. Ubiquilin was originally identified as a Presenilin-interacting protein with a potential contribution to early-onset AD [36, 37], but its functional interaction with other AD genes was ultimately demonstrated in transgenic flies. These studies demonstrated that Ubiquilin regulates Psn activity and uncovered its direct interaction with and regulation of APP [38-40]. Finally, recent genetic screens have identified new modifiers of Psn activity that also interact genetically with APP [41, 42]. These observations suggest an unexpected level of complexity in the regulation of APP processing, expanding the number of potential targets for AD therapeutics.

An alternative approach to the production of Aß42 through the amyloidogenic pathway is to directly express Aß42 fused to a signal peptide for secretion in flies [43-46]. Aß42 induces strong phenotypes in several assays, including memory and learning deficits, making this model very attractive for uncovering the genetic mechanisms of Aß42 neurotoxicity, and for testing drugs with protective activity. Given the relative ease to generate transgenic animals, different forms of the Amyloid-ß peptide, including Aß40, Aß42, Aß42 with the familial AD Arctic mutation, and Aß42 with artificial mutations, have been compared *in vivo*. Overall, these studies confirmed experimentally that Aß42 aggregation propensity correlates with neurotoxicity; thus, strategies that prevent Aß42 aggregation should reduce toxicity [43, 47, 48]. For instance, feeding flies with Congo Red, a dye that binds to amyloids and prevent Aß42 fibrillization *in vitro*, prevents neurotoxicity [45]. In addition, compounds that stabilize the α-helical conformation of Aß13-26 also rescue Aß42 toxicity in flies [49]. These strategies make *Drosophila* a key tool for *in vivo* testing of promising AD compounds. For more details, comprehensive reviews on AD models in flies are available in the recent literature [50, 51].

Frontotemporal dementias (FTD) refer to a complex group of disorders that present with radical personality changes with involvement of either the frontal or the temporal lobes. FTP are mostly sporadic, but one of its forms, frontotemporal dementia with parkinsonism linked to chromosome 17 (FTDP-17), is associated with mutations in Tau. This connection explains the role of Tau accumulation in other forms of FTD, which collectively receive the name of tauopathies. The first fly model of tauopathy was based on expression of wild type Tau and mutations linked to FTDP-17 (Table **2**) [52]. Human wild type and mutant Tau disrupt the eye, induce locomotor dysfunction and shorten lifespan. Similarly, expression of bovine Tau fused to green fluorescent protein (GFP) caused axonal degeneration, while fly Tau disrupted the eye [53, 54], supporting the key role of Tau in neuronal homeostasis. Additionally, flies contributed to the identification of Actin cytoskeleton as modifiers of Tau-induced eye phenotype [55, 56]. The neurotoxicity of Tau-R406W is associated with accumulation of Actin filaments, which is dependent on Tau phosphorylation [57]. Another pathway that received attention in Tau neurotoxicity was cell cycle regulation. Tau-R406W aberrantly induces activation of cell cycle proteins through activation of TOR (target of rapamycin), which, in turn, mediates Tau neurotoxicity [58]. Finally, several studies on Tau have identified the kinases that phosphorylate Ser and Thr residues, and the consequence of phosphorylation on Tau conformation and aggregations. This is discussed in more detail in section 8.

### 4.2 Parkinson’s disease

Parkinson’s disease is the most prevalent movement disorder, affecting four to six million patients worldwide. PD patients suffer from tremors, rigidity, bradykinesia (slow movements), and difficulty in balance, symptoms associated with the loss of dopaminergic neurons in the substantia nigra and locus ceruleus of the brain [59]. The affected neurons are characterized by cytoplasmic protein aggregates known as Lewy bodies and Lewy neurites, which are enriched with α–Syn. However, other proteins such as ubiquitin and heat shock chaperones are also present, suggesting that these neurons may suffer from abnormal protein quality control mechanisms [59, 60]. Although most PD cases are sporadic, genetic defects in rare familial cases have provided valuable insights into the pathogenesis of PD. Notably, three point mutations in α–Syn (A30P, E46K and A53T) are associated with autosomal dominant forms of PD [60]. Allele multiplication of *SNCA*, the gene encoding α–Syn, also cause familial PD, suggesting that excess of wild type α–Syn can lead to disease [60]. Inherited forms of PD have been associated with at least 11 other loci, including Parkin, DJ-1, PINK1, and LRRK2/Dardarin (reviewed in [61]). Due to space limitations, we will focus on α–Syn proteinopathy in flies. For specialized reviews on fly models involving other familial PD genes see [62, 63].

α–Syn is a soluble synaptic protein that has been recently proposed to exist natively as a helical tetramer (Bartels* et al, *Nature, 2011). α-Syn aggregates easily *in vitro* and its oligomers are very toxic to cultured neurons. However, α–Syn misexpression does not induce neurotoxicity in transgenic mice, leaving a big question about the role of α–Syn in the neurodegenerative cascade of PD. When Feany and Bender showed that normal and mutant forms of α–Syn (Table **3**) induce selective degeneration of dopaminergic neurons, the paper was warmly welcomed [9]. These flies also showed locomotor dysfunction as well as α–Syn-rich cytoplasmic inclusions reminiscent of Lewy bodies, recapitulating essential features of PD. However, other models developed independently showed reduced α–Syn neurotoxicity: Bonini and colleagues reported a 50% reduction in dopaminergic cells [64], while other authors reported even lower penetrance [65]. Differences in the experimental approaches seem to explain these discrepancies [62, 63]. New methodologies, including semi-automated quantification of dopaminergic neurons and increased α–Syn expression through optimized translational efficiency, recently confirmed α–Syn toxicity in transgenic flies [66, 67]. Additionally, structure-function studies identified α–Syn regions that play a role in aggregation and neurotoxicity in flies [68], while α–Syn variants generated by rational design demonstrated that mutants with impaired ß-state structure (surprisingly) exhibit higher neurotoxicity in flies [69]. Based on these observations, *Drosophila* seems to be the best *in vivo* model of α–Syn neurotoxicity available, making it a powerful tool for gene and target discovery.

### 4.3 Huntington’s Disease and other polyQ Disorders

Polyglutamine (polyQ) diseases are devastating and incurable neurodegenerative conditions with autosomal dominant inheritance and worldwide distribution. At least nine polyQ disorders have been described, including HD, spinobulbar muscular atrophy (SBMA), dentatorubral-pallidoluysian atrophy (DRPLA) and six Spinocerebellar ataxias (SCA1, 2, 3, 6, 7 and 17) [70]. PolyQ disorders are linked to the expansion of a glutamine-coding CAG repeat within the open reading frame of nine unrelated genes. The polyQ expansion perturbs protein stability leading to misfolding, aggregation, inclusion formation and extensive neurodegeneration. Although these nine polyQ proteins are broadly expressed in the CNS, neurodegeneration occurs in selective regions of the brain, resulting in clinically distinct neuropathologies. In all these disorders, the mechanisms of neuronal cell death are largely unknown and probably involve a variety of gain-of-function activities. To help understand these issues, several *Drosophila* models of polyQ disorders have been generated (Table **4**). For a more comprehensive review of these models, specialized reviews are available [63, 71].

*Huntington’s disease*. HD is the most common polyQ disorder affecting one in 10,000 individuals, and is characterized by involuntary movements (chorea), psychiatric disturbances, dementia, and premature death [72]. HD is caused by a CAG expansion (>36) within exon 1 of the *Huntingtin* (*Htt*) gene. The first *Drosophila* model that recapitulated relevant features of HD pathogenesis was reported in 1998 [5]. These flies expressed an N-terminal fragment of human Htt with variable lengths of polyQ tracts under the control of an eye-specific promoter, which led to nuclear inclusions and age- and polyQ length-dependent degeneration of photoreceptor neurons. Since manipulation of these Htt transgenes was restricted to the fly eye, a subsequent model expressing a mutant N-terminal fragment (Htt-ex1) capitalized on the flexibility of the UAS/Gal4 system [73]. Over the last decade, additional models that express different N-terminal fragments or full-length Huntingtin under control of the UAS sequence have appeared (Table **4**). Overall, these models have made important contributions to understanding the molecular pathology of the disease: Htt aggregates sequester other expanded polyQ proteins in the cytoplasm, leading to disruption of axonal transport [30, 74], while SUMOylation and ubiquitination of the expanded Httex1p regulate its neurotoxicity [75]. A new disease mechanism was revealed with the full-length Htt fly model, consisting in Ca^2+^-dependent increase in neurotransmitter release efficiency, which occurs even before expanded Htt is imported into the nucleus [76]. Several other contributions of HD models in gene and drug discovery are discussed below.

*Spinocerebellar ataxias (SCAs).* The six known polyQ-related SCAs (1, 2, 3, 6, 7 and 17) are the most common cause of dominantly inherited ataxia, accounting for over 50% of ataxia patients worldwide [70]. The clinical features of ataxias reflect damage to the cerebellum; however, many SCAs are characterized by their extracerebellar brain involvement [70]. SCA1 and SCA3 fly models were among the first reported [4, 6]. What made them particularly attractive despite being rare disorders is that *(a)* SCAs exhibit a purely genetic inheritance pattern and *(b)* the full-length protein (rather than a proteolytic fragment) is involved in pathogenesis, which facilitated construct design. SCA1 and SCA3 have played a key role in the discovery of disease relevant mechanisms that are described below. 

*PolyQ-only models*. Fly models that express pure polyQ repeats revealed that expanded polyQ tracts alone are intrinsically cytotoxic, form aggregates, and lead to neurodegeneration and premature death [7, 8]. Interestingly, polyQ toxicity was neutralized by protein context [7]. This model was also used to identify genetic modifiers of polyQ neurotoxicity [8]; however, given the influence of the protein context, most laboratories prefer to use disease-related polyQ-containing proteins.

### 4.4 Amyotrophic Lateral Sclerosis

ALS is an incurable, devastating neurodegenerative disorder with a rapid disease course. ALS affects 5 out of every 100,000 people worldwide and is characterized by the selective death of upper and lower motor neurons, causing progressive muscle atrophy, paralysis, and death within one year of diagnosis. Approximately 5-10% of ALS cases are familial (fALS), and 20% of these patients carry mutations in the gene encoding Cu/Zn superoxide dismutase 1 (SOD1), a ubiquitous cytosolic enzyme that protects against toxic superoxide radicals. Out the 153 amino acids of SOD1, more than 125 distinct mutations have been linked to fALS [77]. The mechanisms involved in motor neuron death are largely unknown; current hypotheses for ALS pathogenesis include oxidative damage, accumulation of intracellular SOD1-positive aggregates, mitochondrial dysfunction, axonal transport defects, and astroglial cell pathology, to name a few [78].

Modeling SOD1-dependent pathology in flies has mirrored some of the difficulties experienced by mouse laboratories. For instance, overexpression of wild type hSOD1 in motor neurons dramatically extended lifespan, whereas expression of the fALS mutant hSOD1-G41S was not detrimental (Table **3**) [79, 80]. Moreover, expression of human wild type and mutant SOD1 under the control of endogenous *dSod* regulatory sequences failed to induce neuronal dysfunction in *dSod* null flies [81]. These results are consistent with the lack of motor neuron degeneration upon neuron-specific expression of mutant SOD1 in transgenic mice. Since SOD1 is expressed ubiquitously in the CNS, other cell types such as astrocytes may play a role in neuronal degeneration (non-cell-autonomous toxicity), explaining the difficulties in modeling SOD1 neurotoxicity in animal models [77]. More recently, another fly model of ALS showed that wild type and mutant hSOD1, but not dSOD1, induced locomotor dysfunction with abnormal synaptic transmission and aggregation of hSOD1, but no apparent neuronal loss [82]. Other fly models for non-SOD1 fALS, including those expressing TAR DNA-binding protein 43 (TDP43) and vesicle-associated membrane protein B (VAPB), are extensively reviewed elsewhere [63].

### 4.5 Prion Diseases

Prion diseases (PrD) are unique among the protein misfolding disorders because they can present with sporadic, genetic, and infectious etiologies. These neurodegenerative disorders affect humans and other mammals and lead to dementia, motor dysfunction, and, eventually, death [83]. The Prion protein (PrP) is a membrane-anchored glycoprotein highly enriched in the brain that has an essential role in the pathogenesis of PrD [84]. PrP undergoes irreversible conformational changes from the cellular, α–helix-rich isoform (PrP^C^) to pathological, ß–sheet-rich isoforms (PrP^Sc^) that are partially resistant to protease degradation. It is well accepted that the PrP^C^ to PrP^Sc^ conversion is a key event leading to aggresive spongiform neurodegeneration and death. Unfortunately, major gaps exist in our understanding of how the conformational conversion of PrP occurs and how it ultimately kills neurons. 

The development of *Drosophila* models of PrD has been particularly challenging (Table **3**). The first two attempts to induce PrP-dependent neuropathology resulted in no obvious degenerative effects (reviewed in [85]). These models seemed to accumulate low levels of PrP and led the authors to conclude that flies were inadequate for studying PrP biology. Later on, flies expressing a different PrP mutant associated with a genetic PrD were found to induce brain degeneration associated with PrP aggregation [86], and altered synaptic architectures in larval neuromuscular junctions [87]. However, aged flies did not accumulate detergent-insoluble or protease-resistant PrP conformers, thus missing two hallmark features of pathogenic PrP.

In sporadic Creutzfeldt-Jakob disease (CJD), which accounts for 85% of all CJD cases in humans (by far the most common PrD), spontaneous misfolding of wild type PrP is responsible for disease [88]. To gain insights into the mechanisms involved in the spontaneous misfolding of PrP *in vivo,* we created flies expressing wild type PrP from hamster (HaPrP). The hamster is an excellent model for PrD because it undergoes aggressive disease progression with high amounts of pathogenic PrP conformers, suggesting that HaPrP is highly prone to populate the ß-state. In young flies, HaPrP exhibits properties of native PrP^C^; in aged flies, HaPrP acquires PrP^Sc^–like properties, such as resistance to denaturing agents and immunoreaction with PrP^Sc^-specific conformational antibodies, although it is not resistant to proteases. The accumulation of these PrP^Sc^–like conformers correlate with severe spongiform degeneration in brain neurons, indicating that the prototypical, infectious PrP^Sc^ conformer is not required to induce spongiform degeneration [89]. To further understand how the primary amino acid sequence of PrP determines its structural dynamics, we created transgenic flies expressing wild type PrP from rabbit (RaPrP), an atypical mammal that is resistant to prions [90]. RaPrP does not accumulate in PrP^Sc^–like conformations and is not neurotoxic, suggesting that protective amino acid substitutions prevent the population of the ß-state [90]. A recent paper identified a RaPrP-specific hydrophobic staple that links a loop to helix 3 and increases the conformational stability of the globular domain [91, 92]. However, *in vivo* evidence for the protective activity of this and other residues is still missing, creating an ideal niche for *Drosophila* research. This type of function/structure analyses will help to better understand the rules governing PrP misfolding and pathogenesis.

## 5 MODELING PROTEINOPATHIES IN FLIES: MAKING EYE CONTACT 

**Fig. (2):**
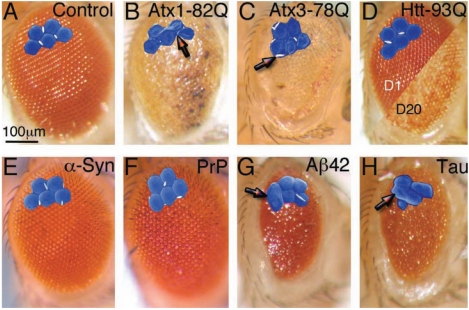
**Disruption of the fly eye by expression of amyloidogenic proteins.** (**A**-**H**) Microphotographs of the *Drosophila* compound eye. Higher magnifications show a detail from scanning electron microphotographs (artificially colored). (**A**) Control eye showing normal pigmentation and organization of the ommatidia. (**B**) Flies expressing Atx1-82Q show highly disorganized (glassy), depigmented eyes, with necrotic spots. High magnification shows poorly differentiated ommatidia with small bristles (arrow). (**C**) Flies expressing Atx3-78Q show disorganized and depigmented eyes with normal bristles. (**D**) Flies expressing Htt-93Q have normal eye structure at day 1 (left), but the underlying retina degenerates by day 20, resulting in patchy depigmentation (right). (**E** and **F**) Flies expressing 3 copies of wild type α-Syn (**E**) or wild type hamster PrP (**F**) have normal eye structure and pigmentation. (**G** and **H**) Flies expressing Aß42 (**G**) or wild type Tau (**H**) have small, disorganized, bumpy eyes resulting from fusion of ommatidia (arrows).

The pathogenesis of the proteinopathies involves gain-of-function mechanisms by either wild type or mutant forms of the human protein. Therefore, misexpression of the disease-related proteins in flies should be highly toxic to brain neurons, but also (potentially) to other tissues and cell types. Following this rationale, several laboratories expressed these proteins in different tissues of the peripheral nervous system, including the eye and the sensory bristles. The compound insect eye has a highly organized lattice that is very sensitive to genetic disruption of various biological processes, can be easily analyzed under a standard stereoscope, and is non-essential for viability. Expression of some disease-related proteins in the eye leads to disorganization of the eye lattice or rough eye phenotypes, while other proteins do not affect the eye (Fig. **2**). These phenotypes have been very useful as the basis for large scale, unbiased genetic screens and for testing candidate genes (see section 8). Critics may argue that the rough eye does not adequately reproduce the neurodegeneration of brain neurons in the human disorders; thus, the mechanisms associated with protein misfolding toxicity in the eye would be irrelevant to understanding the disease. We can deflect these critiques with several arguments. *(1)* While some disease-related proteins induce a rough eye (Atx1-82Q, Atx3-78Q, Aß42, wild type Tau), others do not (Htt-93Q, α-Syn, PrP, SOD1) (Fig. **2**). Neither gene overexpression nor cellular overload explains photoreceptor toxicity; therefore, *specific pathways* must be perturbed to disrupt the eye. *(2) *Another important observation is that when these disease-related proteins affect the eye, they induce very different phenotypes: disorganized lattice, glassy surface, fused ommatidia, lost pigment, or reduced size (Fig. **2, B,C,D, G** and **H**). Thus, each protein interferes with different pathways/networks in the eye that explain the specific eye perturbations. *(3)* Interestingly, Aß and Tau show similar rough eye phenotypes, with reduced size and fusion of ommatidia (Fig. **2, G** and **H**), suggesting that these two AD genes affect similar pathways/networks during eye development. *(4)* Although mutant Htt does not induce rough eye like mutant Atx1 and Atx3, mutant Htt induces progressive degeneration of the underlying retina. This observation supports the relevance of the protein context in polyQ diseases. Finally, *(5)* the rough eye induced by Aß42 is the same whether Aß42 is expressed in photoreceptors (neuron only) or in all eye cell types, suggesting that the rough eye is a consequence of Aß42 toxicity on photoreceptor neurons, not on the neighboring support cells [46]. Regardless of the advantages of using the rough eye for rapid screening purposes, it is advisable to develop assays that support neurotoxicity to central neurons for secondary screens or validation purposes.

Another common approach in fly models of proteinopathies is to use locomotor activity to assess neuronal dysfunction. In fact, all the models described here show progressive locomotor dysfunction, including the wild type alleles of α-Syn, hSOD1, and hamster and mouse PrP. The typical locomotor test measures the ability of flies to climb upward following their innate negative geotaxis, a simple assay easy to set up in the lab, although complex software packages can also analyze more comprehensive behavioral tasks. These locomotor tests are highly relevant to the pathology of movement disorders such as PD, ALS and HD, but they have also been used in models of dementias (AD, PrD). How is this locomotor assay relevant to understand the molecular mechanisms of Aß42 neurotoxicity, a peptide that targets primarily the hippocampus and the cortex? Although Aß42 induces progressive memory deficits in flies [43], these assays are complex and time consuming. Thus, we use locomotor dysfunction as surrogate for *neuronal dysfunction*. Locomotor dysfunction informs us about the ability of any protein to interfere with basic neuronal processes, including cell survival/ apoptosis, oxidative stress and mitochondrial metabolism, cytoskeleton and axonal transport, synaptic architecture and transmission, etc. These cellular activities are essentially the same in all neurons; thus, a locomotor phenotype is an easy indicator of a serious neuronal pathology. In addition to being easy, locomotor assays provide complex information about the underlying cellular pathology. Curiously, Aß42 and Htt induce distinct locomotor phenotypes: whereas flies expressing Aß42 are unremarkably slow, the HD flies exhibit highly uncoordinated movements and shaking, a phenotype reminiscent of the chorea of HD patients. Coincidence? Unlikely. In fact, this locomotor dysfunction indicates that flies undergo specific neurodegenerative changes related to HD pathology, making them an excellent system to understand the molecular mechanisms underlying HD neurodegeneration. The experimental flexibility provided by the locomotor assays allows for fast and efficient characterization of new disease models and of potential genetic and pharmacologic modifiers of the pathology. Many other assays are available for characterizing protein neurotoxicity, including memory and learning, longevity/survival, and analyses of specific neuronal loss such as dopaminergic neurons in PD models. These assays have different applications based on their technical complexity: gene discovery efforts require easy-to-score phenotypes (rough eye), while testing protective genes or drugs demand higher specificity (memory tests, survival of dopaminergic neurons). In any case, the availability of multiple assays is a testament to the flexibility of *Drosophila*. 

## 6 PROTEIN AGGREGATION AND THE NEUROTOXIC PARTICLE

Another key feature of these proteins is their ability to form disease-specific assemblies with characteristic cellular distribution. So far, *Drosophila* models have been very good at reproducing disease-specific protein dynamics: mutant Atx1, Atx3, and Htt accumulate in nuclear inclusions (NI) [4-6], whereas both wild type and mutant α-Syn accumulate in Lewy body-like cytoplasmic aggregates [9]. In addition, wild type PrP accumulates in detergent-soluble aggregates in lipid rafts, although some of it is retained in the secretory pathway as a consequence of early misfolding [89]. Overall, all of these proteins accumulate and aggregate in the right subcellular compartment and lead to neurotoxicity, making these models powerful tools for understanding *in vivo* protein dynamics and neurotoxicity. However, interesting observations in flies have provided relevant cues about the role of protein aggregates in neurotoxicity. Although mutant Atx1, Atx3 and Htt-ex1 form NI, these large aggregates may have a protective role as suggested by the increase in aggregate size by co-expression of chaperones and Atx1 [6]. Moreover, the HD-FL model exhibits signs of neurotoxicity without obvious NI, supporting the role of other conformers in neurotoxicity [76]. Also, wild type and mutant Tau accumulate in neurites [52, 93], but flies do not accumulate NFT, the typical AD and FTD aggregates containing hyperphosphorylated Tau [52]. This observation suggests that Tau-dependent neurodegeneration does not require NFT and supports the role of soluble assemblies in toxicity. Similarly, flies expressing HaPrP display extensive vacuolar degeneration without accumulation of the protease-resistant PrP^Sc^ isoform, suggesting that different PrP isoforms are responsible for infection and neurotoxicity [89, 94]. In flies, Aß42 aggregates in both extra- and intracellular amyloid aggregates [43], which have also been observed in AD patients [95], despite having strong signal peptides, suggesting that Aß42 is internalized after secretion. Moreover, the intrinsic aggregation propensities of 17 artificial mutations in Aß42 (chosen from 798 variants tested *in silico*) exhibited a strong correlation with their neurotoxicity in flies [47]. The exception was a double mutant with the highest predicted aggregation propensity (E22G, I31E). It turns out that this double mutant rapidly formed fibrillar aggregates, a state less toxic than soluble oligomers and protofibrils. This study demonstrated that Aß42 neurotoxicity is tightly regulated by its fibrillation, and that both too little and too much aggregation could reduce Aß42 neurotoxicity. Overall, *Drosophila* models of proteinopathies have provided important clues for uncovering the connection between protein aggregation and neurodegeneration.

## 7 LIMITATIONS: THE ELEPHANT-SIZE FLY IN THE ROOM

Disclaimer: Fly models of human diseases are just that, models. The anatomy and physiology of invertebrates are significantly different from those of humans, which impose constraints on translatable research. In the case of neurodegenerative diseases, an obvious limitation of fly models is the reproduction of physiological disease conditions: fly models cannot replicate the decades-long progression of the disease nor the regional- and neuronal-specific brain pathology. Moreover, fly models are based on gene misexpression, in which transgenes are not regulated by endogenous promoters. These critiques are legitimate, but the same could be said about many popular mouse models based on transgene overexpression (e.g., SCA1-B05, HD-R6/2, AD-Tg2576). Although several knock-in mouse models with late onset and slow progression have been generated in the last decade, they have not eliminated the use of misexpression models because the latter have more robust phenotypes and are easier to use for discovery purposes. Likewise, *Drosophila* models are still relevant towards uncovering disease mechanisms, target discovery, and other innovative applications (see below). 

But, is the fly still a relevant model of neurodegenerative disorders? Has it become unfashionable? The evidence says no: the number of publications continues on an upward trend (Fig. **1**) and many of those papers continue to appear in high-quality journals. However, both public and private funding agencies seem reluctant to fund studies in *Drosophila*, moving away from basic or exploratory research projects. This may be a shortsighted approach since some of the most spectacular discoveries with human health applications have come from basic research (RNAi in worms, circadian rhythm in flies). Ultimately, it is our responsibility to focus our research towards relevant, innovative, and/or translational areas, and to explain better the relevance of our research to the general public. 

## 8 ROBUST PHENOTYPES LEAD TO GENETIC SCREENS

Fly models of proteinopathies exhibit neuronal dysfunction, neuronal loss, and protein aggregation. These phenotypes have been described in many disease models, including mice and cultured cells. What, then, makes *Drosophila* relevant? Without a doubt, *Drosophila* stands out because of the ability to perform genetic screens for modifiers of neurotoxicity in a cost- and time-efficient manner. Fruit flies are an excellent model system for *in vivo* testing of small sets of interesting genes (candidate approach) by taking advantage of existing loss-of-function or overexpression alleles, or by creating new transgenic flies in less than 10 weeks. Moreover, flies have also been used for unbiased screens of large sets of mutants that allow the identification of new genes and molecular pathways involved in disease pathogenesis. Here, the eye phenotypes have played a fundamental role because the easy analysis of the rough eye under the dissecting microscope allows testing of hundreds of genes per week and over a thousand genes per month. Other phenotypes such as reduced survival or locomotor dysfunction are also compatible with large genetic screens, although they are more time-consuming and may require the use of advanced engineering and software. The more time-consuming the task, the fewer conditions (genes) can be tested. That explains why the largest modifier screens have been performed in the eye [6, 55, 96, 97]. These are a few examples of the contributions of fly genetics to uncovering disease mechanisms.

*Coping with protein aggregation: Hsp70*. Several amyloidogenic proteins aggregate inside the cell, suggesting a role for protein quality control mechanisms in pathogenesis. Cell culture studies demonstrated the role of molecular chaperones, particularly the Heat shock protein 70 (Hsp70), in Atx1 aggregation and neurotoxicity [98]. However, *in vivo* evidence for this protective activity was missing. N. Bonini showed that overexpression of an inducible isoform of human Hsp70 suppressed the rough eye phenotype and Atx3 aggregation in flies [99]. In an unbiased genetic screen, we found that the Hsp70 co-chaperone Hsp40 suppressed the rough eye of Atx1 [6]. This ability of molecular chaperones to suppress pathogenic protein aggregation and neurotoxicity was later expanded to include other disease models. For instance, co-expression of Hsp70 and α–Syn alleviated dopaminergic neuronal loss, while disruption of endogenous chaperone function enhanced neuropathology [64]. Not surprisingly, Hsp70 later showed the ability to block Atx1 and α–Syn aggregation in transgenic mice [100, 101]. Trying to extend this protective activity to extracellular amyloids, we next wondered if Hsp70 could also reduce misfolding of HaPrP, a membrane anchored protein. Interestingly, in flies expressing HaPrP, Hsp70 accumulated in the lipid rafts (a highly specialized membrane domain) and reduced misfolding and neurotoxicity of HaPrP, uncovering a new ability for Hsp70 in preventing misfolding of an extracellular protein [89]. These observations illustrate the value of the chaperone system and its implications in the design of rational therapeutic approaches for several proteinopathies (see below).

*Protein misfolding in the ER: XBP1*. Protein misfolding in the ER and the secretory pathway induces the unfolded protein response (UPR), which differs significantly from the response to misfolded proteins in the cytoplasm and nucleus. The UPR consists in the activation of three independent pathways (PERK, IRE1 and ATF6) that function to reduce protein synthesis and elimination of misfolded proteins in the ER. One of the UPR branches consists on the ER stress sensor IRE1 and its downstream effector X-box binding protein 1 (XBP1), a transcriptional regulator that induces the expression of ER chaperones and other factors that reduce protein misfolding in the ER [102]. AD brains show signs of ER stress, as indicated by the activation of two sensors and several downstream effectors; however, the protective role of XBP1 and other ER stress-response pathways in AD had not been demonstrated *in vivo*. We recently described that flies expressing a bi-cistronic Aß42 construct induce ER stress and activation of XBP1 [46]. Moreover, XBP1 overexpression rescues and XBP1 loss-of-function enhances Aß42 rough eye phenotype. This protective activity was mediated by the reduction in ryanodine calcium channels in the ER, thus preventing the release of pro-apoptotic levels of calcium in the cytoplasm. Interestingly, many proteinopathies activate ER stress regardless of the localization of the misfolded conformers, suggesting that ER homeostasis may be affected during neuronal degeneration [103].

*Posttranslational modifications: Phosphorylation*. A recurrent theme in proteinopathies is the role of post-translational modification, in particular phosphorylation, in protein aggregation and neurotoxicity. The AD brain contains NFT in which Tau is hyperphosphorylated. Tau is a long protein with 79 putative Ser/Thr phosphorylation sites, and Tau phosphorylation increases aggregation and recognition by disease-specific, conformational antibodies. *Drosophila* has played a key role in the *in vivo* determination of many phosphorylation sites. Genetic analysis demonstrated that Cdk5 and GSK-3 were Tau kinases that regulate Tau aggregation and neurotoxicity [55, 93]. Also, modification of the phosphorylation sites showed their differential contribution to Tau aggregation and neurotoxicity. Whereas the Ser-Pro/Thr-Pro sites seemed to work in concert (mutating up to five sites had no significant effect, but mutating all 14 sites dramatically increased Tau aggregation), the non Ser-Pro sites S262 and S356 played critical roles in Tau aggregation by promoting the phosphorylation of Ser-Pro/Thr-Pro sites [57, 104-107]. Along the same line, Atx1 and α–Syn phosphorylation also proved relevant in neuropathology. *In vitro *evidence indicated that Atx1 phosphorylation by Akt1/PKB at S776 promoted binding of 14-3-3, aggregation, and neurotoxicity [108]. Reduced Akt1 function suppressed the rough eye of Atx1 flies, while a S776A substitution in mice reduced Atx1 toxicity, demonstrating the functional relevance of Atx1 phosphorylation [108, 109]. Finally, α–Syn phosphorylation at S129 and T125 exhibited opposing effects, the first being neurotoxic and the second neuroprotective, suggesting that α–Syn toxicity depended on a balance between these two modifications [110, 111]. Thus, these studies played a critical role in identifying how phosphorylation contributes to the generation of the neurotoxic isoform, a key piece of information for the rational design of neuroprotective compounds.

*Unbiased screens*. If candidate pathways have found in *Drosophila* the ideal *in vivo *system for fast functional tests, unbiased genetic screens have expanded our understanding of the molecular mechanisms underlying protein misfolding and neurodegeneration. Genetic screens provide the means for unbiased gene discovery without prior knowledge of the involved pathways. Seminal contributions include the confirmation that protein quality control mechanisms, including molecular chaperones and protein degradation, play important roles in polyQ neurotoxicity [6]. More importantly, genetic screens served a unique role towards identifying novel pathways implicated in Atx1 and Atx3 neurotoxicity, including RNA metabolism, cellular detoxification, and transcriptional regulation [6, 96]. Not surprisingly, several kinases and phosphatases were identified as modifiers of Tau toxicity in the fly eye [55]. On the other hand, genetic modifiers of Aß42 implicated the secretory pathway, cholesterol homeostasis and copper transport in its neurotoxicity [97]. Thus, four genetic screens based on gene misexpression using the same collection of random insertions identified very different modifiers, revealing the unique mechanisms of neurotoxicity activated in each model. In addition, a loss-of-function screen identified the role of innate immunity and inflammation pathways in Aß42 neurotoxicity [112]. By testing collections of genetic modifiers of one model against other models, common and distinct mechanisms of pathogenesis can be identified [55, 97, 113]. Other key findings show that polyQ expansions in Atx1 contribute to gain- and loss-of-function mechanisms by specific promotion/disruption of endogenous protein complexes [114]. Interestingly, interaction of Atx1 and Atx3 with other proteins bearing polyQ revealed that the activity of Atx2 (SCA2) is critical for SCA3 and SCA1 pathogenesis [115, 116]. A comprehensive list of genetic pathways and modifiers of polyQ diseases can be found elsewhere [117].

*Complex screens*. Important contributions to the field of target discovery have come from combining high throughput screening (HTS) in a variety of platforms with *in vivo* validation in flies. For instance, integration of a yeast two-hybrid screen and affinity pull-down assays with genetic manipulation of an HD fly model led to the identification of several Htt-interacting proteins that were known modifiers of neurodegeneration [118]. Another screen combining biochemical approaches with fly genetics identified three matrix metalloproteases as modifiers of Htt proteolysis and toxicity [119]. More recently, genome-wide RNAi screens for modifiers of Htt aggregation *in vitro* combined with validation in fly models, identified new regulators of Htt aggregation and toxicity, including genes related to nuclear transport, nucleotide processing, and signaling [120, 121]. A different approach consists in identifying aberrant pathways through gene expression analyses (microarrays). Expression of Aß42 in flies led to significant changes in the expression of oxidative stress genes and genetic tests confirmed the role of the iron-binding protein Ferritin and Catalase as strong suppressors of Aß42 neurotoxicity [122]. Given the lack of robust phenotypes in α–Syn flies, genetic screens have not been possible with this model. However, combination of a primary genetic screen in a yeast model of α–Syn aggregation with subsequent validation in a fly model of PD led to the identification of Rab guanosine triphosphatase 1 (Ypt1p/Rab1) as a strong suppressor of dopaminergic cell death [123]. This suppression was further confirmed in primary cultures of mammalian dopaminergic neurons, highlighting the value of simple model systems in the gene discovery process. 

## 9 THE FLY PHARMACY 

During the last 20 years, drug research has experienced dramatic changes resulting from newer technologies that include genomics and proteomics, structural biology, informatics, automated imaging, microfluidics, and robotics. Biotechnology companies have combined these emerging technologies to design robust HTS methods for drug discovery. Despite these technological advances, effective new drugs come out at a slow rate due to three major limitations: *(1)* current HTS methods rely mostly on the availability of a short list of validated targets; *(2) *inability to reproduce human pathogenic mechanisms *in vitro*; and *(3)* inefficacy of hits when tested in rodent models. To bypass these limitations, efforts have been directed to screen chemical libraries in simple model organisms, where complex biological processes can be studied in the context of intact living systems. In this regard, *Drosophila *holds tremendous potential in pharmacological research [124, 125]. A considerable amount of FDA-approved drugs act on receptors or downstream effectors of major signal transduction pathways conserved in flies and humans, including those for Wnt/wingless, Hedgehog, TGF-ß, Notch, Ras/MAPK and insulin. The availability of several fly models of human diseases may facilitate testing of large compound libraries for the few hits that can revert, prevent, or delay neurotoxicity. Here, we provide a perspective on how *Drosophila* can complement ongoing efforts in drug development.

*How to drug a fly*. Compounds can be easily provided to larvae or adult flies by mixing them with the food or in a sugary solution placed in a small Whatman paper from which starved flies will drink. Using these strategies, administration of 4-phenylbutyrate throughout adulthood extended lifespan, while compounds that alter dopamine levels modified aggressive behaviors [126]. In other cases, vaporized chemicals such as cocaine or ethanol allowed the simultaneous treatment of large groups of flies, making this method suitable to automation and HTS [126]. However, determination of the actual inhaled dose and the vaporization of active compound can be limiting factors. An alternative approach, which offers better control over administration of pharmacological compounds, is direct intra-abdominal or intra-thoracic injection [127]. Although this procedure requires advanced skills and is more laborious, it allows a precise delivery of compounds under the microscope. Interestingly, microfluidic devices coupled with computer-controlled injection systems have been recently developed to inject fly embryos [21, 22]. These efforts emphasize the interest of the *Drosophila* community for achieving *bona fide* HTS conditions.

*From genes to targets: the hit list. Drosophila* models of proteinopathies have contributed to the development of novel therapeutic approaches [71]. For instance, a seminal work reported that the polyQ domain of Htt directly binds the acetyltransferase domains of Creb-Binding Protein and p300/CBP-associated factor, resulting in reduced histone acetylation and changes in gene expression. To compensate for this, flies were treated with histone deacetylase (HDAC) inhibitors (trichostatin A and suberoylanilide hydroxamic acid), which reversed Htt neurotoxicity [73]. Other important contributions to the suppression of Htt neurotoxicity include the mTOR inhibitor rapamycin (autophagy) [128], the inhibition of transgulatminase 2, a transcriptional repressor overexpressed by mutant Htt, with ZDON [129], ERK activation by polyphenols [130], and Meclizine, an FDA-approved drug with anti-histaminergic activity, that also suppresses mitochondria respiration and reduces oxidative stress [131]. Inhibitors of Sirtuin 2, a member of the histone deacetylase family of proteins, rescued α–Syn-dependent loss of dopaminergic neurons [132] and also showed neuroprotection in Htt flies [133]. Another example of the gene-to-drug transition is the rescue of Atx3 and α–Syn toxicity by Hsp70. The pharmacologic strategy to stimulate Hsp70 expression consists on inhibiting Hsp90, which acts as a negatively regulator of the heat-shock response. This approach worked by feeding flies the Hsp90 inhibitor geldanamycin, which suppressed α–Syn neurotoxicity [134]. Since geldanamycin is very toxic, a new generation of Hsp90 inhibitors with improved activity and lower toxicity has been developed, including 17-AAG, with potential uses in cancer and neurodegenerative diseases. Taken together, these studies illustrate the potential of flies in the discovery and validation of neuroprotective compounds.

## 10 * DROSOPHILA* GOES BIOTECH

Several pharmaceutical and biotech companies have embraced fruit flies in their research programs. For instance, Exelixis Inc. created an impressive collection of gene disruption mutants for use in target identification, while Curagen Corporation generated a comprehensive protein interaction database of fly proteins. Similarly, Aktogen Limited aims to accelerate the discovery of drug targets for the treatment of neurodegenerative and neuropsychiatric disorders using flies. Helicon Therapeutics is exploiting an array of proprietary technologies from Cold Spring Harbor Laboratory to identify fly genes that control memory, and Medros grows fruit flies in a standard 96-well format combined with robotics to screen for drugs against human chronic diseases. EnVivo Pharmaceuticals was founded in 2001 to conduct pharmacological screens in fly models of human neurodegenerative disorders using high-speed cameras and a proprietary software known as PhenoScreen. This effort was later transformed into optimized, drug screen services with fly models of neurodegeneration now offered by Vitruvean LLC to pharmaceutical companies and academic labs. Thus, the opportunity to request drug screens with the library of choice may contribute significantly to the drug discovery process in the years to come.

## 11 CONCLUSIONS

*Drosophila* models of proteinopathies have provided some of our earliest insights into the mechanisms leading to neuronal cell death. The ability to manipulate the fly genome in a flexible and time-efficient manner, along with an extensive range of genomic resources and genetic tools, allows the study of neurodegenerative processes at a level of resolution not possible in more complex organisms. Thus, fly geneticists are in a unique position to address relevant questions in neurodegenerative diseases, such as: Which are the molecular mechanisms triggering the accumulation of disease-related conformations? What is the identity of the neurotoxic conformer(s) in each disease? Which are the pathogenic cascades leading to neurodegeneration? How do environmental factors and susceptibility genes contribute to neurodegenerative disorders? The extensive genetic arsenal of *Drosophila*, combined with a century of genetic knowledge, will be instrumental to answer these fundamental questions and to pose new hypotheses in the years to come. It will be interesting to see how new technologies, including next-generation sequencing, proteomics, metabolomics, *in vivo *imaging, and single cell analysis, will be utilized in flies to develop a more integrated understanding of the cellular perturbations induced during neurodegeneration.

On the other hand, *Drosophila* is gaining momentum as a platform for therapeutics. This is because, after spending hundreds of millions on R&D, pharmaceutical firms discover that promising compounds are ineffective in clinical trials or are toxic in humans. Therefore, inclusion of model organisms in the initial stages of drug discovery is crucial to validate hits and exclude compounds with unfavorable properties. Unfortunately, *in vivo *studies are usually performed after lead optimization due to the complexity of rodent manipulation, and the relative high number of preliminary hits. The pharmacological susceptibility of fly models and the technological advances described above illustrate their potential for rapid and inexpensive drug evaluation under HTS conditions. Moreover, the ability to conduct large-scale genetic screens represents a unique opportunity to identify relevant targets. While flies cannot replace the need for testing in rodent models, the studies described here highlight their potential to speed up the drug discovery process. Given the successful multidisciplinary efforts of *Drosophila* researchers to identify neurodegenerative targets and pathways (that would be missed by more conventional approaches), we augur more significant contributions in the next decade. Therefore, the future looks bright for *Drosophila* as an instrument for genetic and pharmacological discovery in neurodegenerative proteinopathies.

## Figures and Tables

**Table 1.** *Drosophila* Resources

Name	Website	Features	Content
Flybase	flybase.org	Genes, alleles, phenotypes, sequence, stocks, images, movies	55,000+ gene records from 500+ Drosophilids
Bloomington Drosophila Stock Center	flystocks.bio.indiana.edu	Collect, maintain, and distribute * Drosophila *strains for research	30,810 stocks, 196,930 lines distributed in 2010
DrosDel	drosdel.org.uk	An isogenic deficiency kit for * Drosophila*	15,166 total possible deletions
Drosophila Genetic Resource Center	dgrc.kit.ac.jp/en	Mutants, aberrations, balancers, insertions	17,140 stocks
Drosophila Species Stock Center, San Diego	stockcenter.ucsd.edu	Diverse array of species	250 species in 1499 stocks
Drosophila Genomics Resource Center	dgrc.cgb.indiana.edu	Cell lines, clones, and vectors	135 cell lines, 1,000,000+ clones
Drosophila RNAi Screening Center	flyrnai.org	dsRNA for cell culture assays, coding and non-coding RNAs	13,900 genes, dsRNA in 62 assay plates
Vienna Drosophila RNAi Center	stockcenter.vdrc.at/control/main	UAS-dsRNA in flies	31,896 strains, 13,142 genes (93% coverage)
Berkeley Drosophila Genome Project	fruitfly.org	Sequence data, clones, stocks, libraries	118.4 Mb genome assembly
MODel organism ENCyclopedia Of DNA Elements (modENCODE)	modencode.org	Functional elements in *C. elegans* and *Drosophila *genomes	Transcriptome, regulatory elements
Textpresso for Fly	textpresso.org/fly	Information extracting and processing for fly literature	Literature on Fly: Title: 43186; Abstract: 36029; Total: 99179
The Interactive Fly	sdbonline.org/fly/aimain/1aahome.htm	Guide to Drosophila development and metazoan evolution	Atlases, gene listings
Drosophila Interactions Database	droidb.org	Protein-protein, TF-gene, miRNA-gene, and genetic interactions	400,031 interactions, 15,201 genes
Drosophila Protein Interaction Map	interfly.med.harvard.edu/index.php	Unbiased interaction map of the proteome based on MS analysis	3,546 interactions

**Table 2.** AD/Dementia Models in Flies

Model	Transgene	Features	Relevance	References
APP	dAPPL, hAPP	Full-length APP	Neural differentiation and axon function	[28, 29]
	APP-swe APPΔCT, APPΔNT	APP KM670/671NL, deletion constructs	PNS development	[135, 136]
	Nicastrin, Aph-11, dPsn, hPS2, APP-C99	In vitro, S2 cells	Description of γ-secretase complex	[31]
	dPsn-WT, N141I, L235P, E280A	Fly γ-secretase activity	APP processing, Aß42 toxicity	[33]
	dPsn+fAD	dPsn with 14 mutations found in familial AD	γ-secretase activity	[137]
	dUbqln, hUBQLN1, hUBQ-8i	Fly and two isoforms of human Ubqln	Regulation of γ-secretase activity	[38-40]
	APP-Gal4, APP-C99-Gal4	γ-secretase activity sensor	Genetic screen of APP-processing factors	[40, 138]
	APP, ADAM10/kuz, dBACE	Fly α-secretase, β-secretase activities	APP processing	[32]
	APP-C99	C-terminal 99 a.a.	APP processing, Aß42 neurotox.	[44]
Aß	Aß40, Aß42	Toxicity of Aß40 vs. Aß42	Aß42 neurotox.	[44]
	Aß42, E22G (arctic), L17P (synthetic)	Mutant Aß42	Aß42 neurotox.	[45, 48]
	Pyroglutamate-Aß42	Posttranslational modification	Aß42 aggregation	[139]
	Aß42x2	Highly expressed	Aß42 neurotox.	[46]
Tau	bovTau-GFP	Tau reporter	Axonal tracing	[140]
	hTau, -R406W, -V337M	WT Tau and FTDP-17 mutations	Tau neurotox.	[52]
	hTau 4R	WT Tau expressed in eye	Tau neurotox.	[93]
	dTau	Fly Tau	Memory deficits	[141]
	hTau 3R, 4R	Tau with 3/ 4 microtubule-binding repeats	Tau neurotox.	[53, 142]
	hTau-R406W+S2A, S202A	Mutant Tau + S262, 356 or S202 > A	Tau phosphorylation and neurotox.	[104]
	hTau-T/S>A	14 T/S individually > A	Tau phosphorylation and neurotox.	[105]
	hTau-AP, -E14	All 14 T/S > A or E	Tau phosphorylation and neurotox.	[58, 106]
	hTau-S2A, -S11A	S at 262 and 356 > A, 11 S/T > A	Tau phosphorylation and neurotox.	[107]
Tau, Aß	Aß, Tau	Tau N4R, Aß42	Aß42, Tau interaction	[57, 143]

**Table 3.** PD and other Proteinopathy Models in Flies

Model	Transgene	Features	Relevance	References
PD	α-Syn-WT, A30P, A53T	fPD	α-Syn neurotox.	[9, 64]
	α-Syn-WT	Comparative studies of α-Syn	Unravels technical issues	[65]
	α-Syn-S129A, S129D, YF	S/Y Phosphorylation mutations	Aggregation and neurotox.	[110, 111]
	α-SynΔ71-82, 1-120, 1-87	Deletion constructs	Aggregation studies	[68]
	α-Syn-WT	Highly expressed	a-Syn neurotox.	[67]
	α-Syn-WT, A53T, A56P	ØC31 integrase constructs	Aggregation studies	[69]
PrD	MoPrP-WT, P101L	GSS	PrP misfolding and neurotox.	[86, 87]
	HaPrP-WT	Sporadic PrD	PrP misfolding and toxicity	[89]
	RaPrP-WT	Resistant PrP	PrP misfolding and neurotox.	[90]
ALS	SOD1-WT, G41S	fALS	Longevity studies	[79, 80]
	SOD1-WT, AV4, G37R, G41D, G93C, I113T	fALS *dSod* promoter	Longevity studies	[81]
	SOD1-WT, A4V and G85R	fALS UAS promoter	Neuronal dysfunction	[82]

**Table 4.** Polyglutamine Disease Models in Flies

Model	Transgene	Features	Relevance	References
HD	hHtt-ex1-2Q, -75Q, -120Q	Expressed in the eye	Htt neurotox.	[5]
	hHtt-ex1-20Q, -93Q	First 90 a.a.	Htt neurotox.	[73]
	hHtt-1-171-18Q, -138Q	First 171 a.a.	Htt neurotox.	[144]
	hHtt-1-336-16Q, -128Q	First 336 a.a.	Htt neurotox.	[113]
	hHtt-1-548-0Q, -128Q	First 548 a.a.	Htt neurotox.	[74]
	hHtt-ex1-18Q, -48Q, -152Q	EGFP fusions	Htt aggregation and neurotox.	[120]
	hHtt-ex1-25Q, -46Q, -72Q, -103Q	EGFP fusions	Htt aggregation and neurotox.	[121]
	hHtt^FL^-16Q, -128Q	Full-length	Htt neurotox.and neurobiology	[76]
SCA1	hSCA1-2Q, -30Q, -82Q	Full-length	Atx1 neurotox.and neurobiology	[6]
SCA2	hSCA2 and dSCA2	Full-length and deletion constructs	Atx2 function	[145]
SCA3	hSCA3-27Q, -78Q	C-terminal fragment	Atx3 neurotox.	[4]
	hSCA3-65Q(NLS), hSCA-77Q(NES)	C-terminal fragment	Atx3 neurotox.and axonal transport	[30]
	hSCA3-79Q	C-terminal fragment	Atx3 neurotox.	[146]
	hSCA3^FL^-27Q, -78Q, -84Q	Full-length	Atx3 neurotox.	[147]
SCA7	hSCA7-10Q, -102Q	First 232 a.a.	Atx7 neurotox.	[148]
DRPL	hAtrophin1-26Q,- 65Q	First 917 a.a	At1 neurotox.	[149]
SBMA	hAndrogen Receptor-112Q	N-terminal fragment	AR neurotox.	[150]
	hAndrogen Receptor-52Q	Full-length	AR neurotox.	[151]
PolyQ	20Q, 127Q	HA tagged	polyQ neurotox.	[8]
	22Q, 108Q	Myc/Flag tagged	polyQ neurotox.	[7]

## ABBREVIATIONS

Aβ42: = Amyloid beta peptide
AD: = Alzheimer’s disease
ALS: = Amyotrophic lateral sclerosis
APP: = Amyloid precursor protein
Atx: = Ataxin
Psn: = Drosophila Presenilin
DRPL: = Dentatorubral-pallidoluysian atrophy
GFP: = Green fluorescent protein
HD: = Huntington’s disease
HTS: = High-throughput screen
Htt: = Huntingtin
PD: = Parkinson’s disease
PrD: = Prion disease
PrP: = Prion protein
SBMA: = Spinobulbar muscular atrophy
SCA: = Spinocerebellar ataxia
SOD1: = Superoxide dismutase 1
Syn: = Synuclein
UAS: = Upstream activator sequences
